# Analysis of *MTHFR* and *MTRR* Gene Polymorphisms in Iranian Ventricular Septal Defect Subjects

**DOI:** 10.3390/ijms14022739

**Published:** 2013-01-28

**Authors:** Seyyed Reza Pishva, Ramachandran Vasudevan, Ali Etemad, Farzad Heidari, Makanko Komara, Patimah Ismail, Fauziah Othman, Abdollah Karimi, Mohammad Reza Sabri

**Affiliations:** 1 Genetic Research Group, Department of Biomedical Science, Faculty of Medicine and Health Sciences, Universiti Putra Malaysia, Selangor 43400, Malaysia; E-Mails: reza_xe54@yahoo.com (S.R.P.); r.vasudevan@monash.edu (R.V.); ali_etemad_c@yahoo.com (A.E.); farzad720@gmail.com (F.H.); k_bsbm@hotmail.com (M.K.); 2 School of Science, Monash University Sunway Campus, Jalan Lagoon Selatan, Bandar Sunway, Selangor 46150, Darul Ehsan, Malaysia; 3 Department of Human Anatomy, Faculty of Medicine and Health Sciences, Universiti Putra Malaysia, Selangor 43400, Malaysia; E-Mail: fauziah@medic.upm.edu.my; 4 Pediatric Infectious Research Centre, Mofid Children Hospital, Shariati St, Tehran 15468, Iran; E-Mail: dr_a_karimi@yahoo.com; 5 Pediatric Heart Centre, Isfahan Medical University, Isfahan 73461, Iran; E-Mail: sabri@med.mui.ac.ir

**Keywords:** *MTHFR*, *MTRR*, polymorphism, congenital heart disease, ventricular septal defect

## Abstract

Ventricular septal defect (VSD) is one of the most common types of congenital heart defects (CHD). There are vivid multifactorial causes for VSD in which both genetic and environmental risk factors are consequential in the development of CHD. Methionine synthase reductase (*MTRR*) and methylenetetrahydrofolate reductase (*MTHFR*) are two of the key regulatory enzymes involved in the metabolic pathway of homocysteine. Genes involved in homocysteine/folate metabolism may play an important role in CHDs. In this study; we determined the association of *A66G* and *C524T* polymorphisms of the *MTRR* gene and *C677T* polymorphism of the *MTHFR* gene in Iranian VSD subjects. A total of 123 children with VSDs and 125 healthy children were included in this study. Genomic DNA was extracted from the buccal cells of all the subjects. The restriction fragment length polymorphism polymerase chain reaction (PCR-RFLP) method was carried out to amplify the *A66G* and *C524T* polymorphism of *MTRR* and *C677T* polymorphism of *MTHFR* genes digested with *Hinf1*, *Xho1* and *Nde1* enzymes, respectively. The genotype frequencies of CC, CT and TT of *MTRR* gene among the studied cases were 43.1%, 40.7% and 16.3%, respectively, compared to 52.8%, 43.2% and 4.0%, respectively among the controls. For the *MTRR A66G* gene polymorphism, the genotypes frequencies of AA, AG and GG among the cases were 33.3%, 43.9% and 22.8%, respectively, while the frequencies were 49.6%, 42.4% and 8.0%, respectively, among control subjects. The frequencies for CC and CT genotypes of the *MTHFR* gene were 51.2% and 48.8%, respectively, in VSD patients compared to 56.8% and 43.2% respectively, in control subjects. Apart from *MTHFR C677T* polymorphism, significant differences were noticed (*p* < 0.05) in *C524T* and *A66G* polymorphisms of the *MTRR* gene between cases and control subjects.

## 1. Introduction

Congenital heart disease (CHD) is one of the most commonly occurring noninfectious diseases and birth defects among children. CHD is a condition whereby there is a malformation in the cardiovascular system whilst the child is still at an embryo stage [1]. The frequency of this disease is around 1 in 100 live births, but this figure is different throughout the world [1]. It constitutes one third of all congenital anomalies and is the primary cause of birth defects leading to infant mortality [2]. Congenital heart defects are constituted of several malformations such as hypoplasia, obstruction defects, septal defect and cyanotic defects [3].

Ventricular septal defect (VSD) is the most common form of CHD occurring in children at a range of 1.56 to 53.2 per 1000 live births [4]. In spite of recent advances in the CHD diagnosis and treatment, experts are yet to fully understand the underlying causes of most CHDs. Several genetic association studies have been conducted by comparing the genetic variations of DNA between cases and control subjects [5]. The commonest forms of human genetic pool variation are the single nucleotide polymorphisms (SNPs), which are responsible for 90% of DNA variation [6]. Homocysteine (Hcy), an intermediary in the production of cysteine from methionine, is a non-essential sulfur-containing amino acid. Both environmental and genetic factors affect plasma homocysteine levels. Low consumption of folate, vitamin B12 and B6, smoking and obesity are said to be environmental factors, while the genetic factors include several variants of enzymes involved in the homocysteine metabolic pathway. One genetic factor, which is the change of alanine to valine caused by *C677T* polymorphism of methylene tetrahydrofolate reductase (*MTHFR*), a thermolabile variant with reduced activity, and has been associated with elevated plasma homocysteine concentrations, especially when there is insufficient folate [7]. Nevertheless, other authors have failed to find a significant relationship in analogous studies where contrasting results were evident [8,9].

Methionine synthase reductase (*MTRR*) is one of the main regulatory enzymes in the homocysteine metabolic pathway. This flavoprotein has an important role in maintaining sufficient levels of activated cobalamin, which acts as a carrier for methyl during the remethylation of homocysteine to methionine. Therefore, a lack of this enzyme may cause hyperhomocysteinemia. Wilson *et al*. found an *MTRR* gene polymorphism that is a “missense” mutation (A66G), which leads to an isoleucine being substituted by a methionine residue at codon 22 [10]. Some researchers identified the A66G polymorphism as a contributing element for high plasma Hcy levels leading to incidents of vascular disease [11–13]. However, other studies have failed to provide evidence that *MTRR* polymorphism is one of the causes of alterations in either plasma Hcy levels and/or vascular disease [14,15]. A study carried out in Ahwaz (Iran) from 1998–2007 found that the mean prevalence of CHD was 12.30 per 1000 live births of the total 3061 instances of live births and an annual prevalence ranging from 7.93 to 17.51 per 1000 live births [16]. The folate metabolism genes were highly studied in various populations [17–22]. Apart from that, transcription factor genes were also commonly studied in relation to coronary artery disease (CAD), CHD and VSD in various populations with conflicting results (Table 1).

The aim of this study was to understand the etiology of the genes responsible for VSD in the Iranian population by investigating the *MTHFR* and *MTRR* gene polymorphisms for the development of VSD in Iranian subjects through a case-control study. To our knowledge, no prior studies have been conducted in Iran regarding the association of *MTHFR* and *MTRR* gene polymorphisms and VSD. Taking this into an account, our main objective was to determine whether the *MTHFR* and *MTRR* gene polymorphisms are risk factors or not for the development of VSD in Iranian subjects.

## 2. Results and Discussion

Methionine synthase is critical for homocysteine metabolism, and methionine synthase reductase is required to maintain methionine synthase in an active state. Both enzymes are associated with frequent polymorphisms that alter the primary structure of the proteins, and both have been subject to extensive analysis of metabolite and disease associations. Studies have suggested that the *MTHFR* and *MTRR* gene polymorphisms have become known as eventual contributors to elevated homocysteine plasma concentrations. Previous reports have reported that those genetic polymorphisms are not consistent in coronary artery disease (CAD) subjects in many populations [11,12,27]. These contradictory findings were suggested as the plausible differences in ethnicities. To our knowledge, there are no other reports on the prevalence of *MTHFR* and *MTRR* gene polymorphisms, and CAD has been reported in Asian population. In this study we examined the association of three SNPs; a common *MTHFR* gene polymorphisms *C677T* (rs1801133), and two *MTRR* gene polymorphisms A66G (rs1801394) and C524T (rs1532268) in Iranian VSD subjects. Homozygosity (TT) for this *MTHFR* single nuclear polymorphism is associated with higher homocysteine levels and lower serum folate concentrations [28,29], than heterozygosity (CT) or wild type genotype (CC), hence it is considered as a genetic risk factor for diseases associated with hyperhomocysteinemia [30,31], although some authors failed to show this interrelation [32,33]. Attempting to confirm the existence of an association between *MTHFR* and CHD, several studies with conflicting outcomes have been published [34–39], later studies, however, do not support the theory of *MTHFR* acting as a risk factor for the development of CHD.

To the best of our knowledge, this is the first study to examine the association of *C677T* polymorphism of the *MTHFR* gene and A66G and C524T polymorphisms of the *MTRR* gene among Iranian VSD subjects. This study was designed to compare the genotypes and allele frequencies of polymorphisms between cases and controls. Case control studies are the most common method used for testing associations of genetic polymorphisms with traits [40]. In addition, this study design was selected because it is inexpensive and relatively quick and suitable for relatively small sample sizes. After excluding participants who did not meet the inclusion criteria, an adequate sample size of 248 subjects (123 cases and 125 controls), which was collected from the Mofid Children Hospital (Tehran, Iran) was included in this study.

### 2.1. Socio-Demographic Characteristics

The percentage of males and females among the VSD cases was 46.3% and 53.7% respectively, whereas the percentage of males and females among the control subjects was 44.8% and 55.2%, respectively, in this study. The mean age of case subjects was 4.51 ± 2.39 whereas the mean age of controls was 5.43 ± 0.51. There was a significant difference of mean group of age between cases and controls (*p* < 0.05).

### 2.2. *C677T* Polymorphism of *MTHFR* Gene

**Figure 1:**
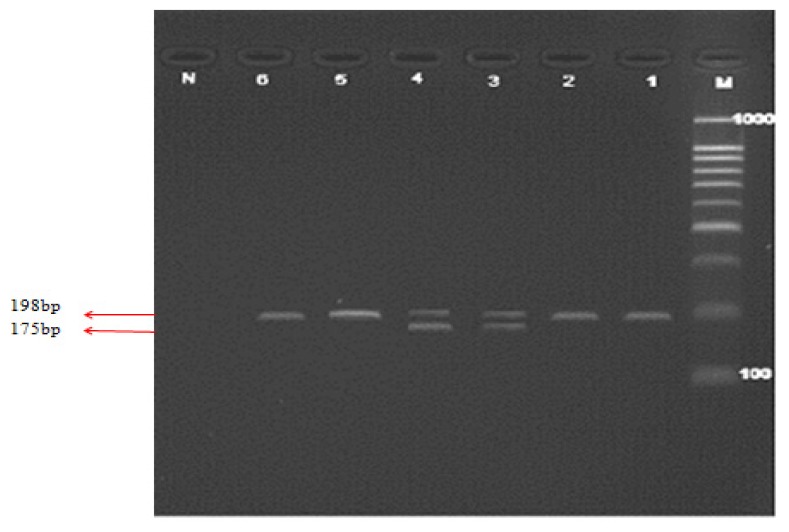
Polymerase chain reaction (PCR) products for methylene tetrahydrofolate reductase (*MTHFR*) *C677T* polymorphism and restricted fragments on 4% agarose gel electrophoresis.

Genotypes of *C677T* polymorphism were determined by incubating the PCR product with *Hinf1* restriction enzyme. The fragments were resolved on 4% agarose gel. Figure 1 shows the PCR product and PCR-RFLP of *MTHFR C677T* gene polymorphism. The genotype and allele frequencies of *C677T* polymorphism of *MTHFR* are shown in Table 2. The Chi-square test did not show any significant differences between genotype and allele frequencies of *C677T* polymorphism among cases and controls (*p* > 0.05) and (*p* > 0.05), respectively. The percentage of CC and CT genotypes among cases was 51.2% and 48.8%, respectively, compared to 56.8% and 43.2%, respectively among control subjects. The derived allele frequencies of C and T alleles were 75.6% and 24.4%, respectively, among cases and 78.4% and 21.6%, respectively, among controls.

Lane M illustrates the 100bp DNA ladder. Lanes 1 and 2 show the PCR products (198bp), Lanes 3 and 4 show the heterozygote fragments (198 and 1475 bp), Lanes 5 and 6 shows the wild type fragment (198 bp) and Lane N represents the negative test control.

Genotype and allele frequencies of *C677T* polymorphism of *MTHFR* were compared between cases and controls. There were no significant differences between genotype and allele frequencies of *C677T* polymorphism among cases and controls (*p* > 0.05). The percentage of CC and CT genotypes among cases was 51.2% and 48.8%, respectively, compared to 56.8% and 43.2%, respectively, among control subjects. The derived allele frequencies of C and T alleles were 75.6% and 24.4%, respectively, among cases and 78.4% and 21.6%, respectively, among controls. As there was no TT genotype in cases and controls, we failed to show an association between *MTHFR C677T* gene polymorphism and the occurrence of VSD. The results obtained from a study done by Zhu *et al.* on a Chinese population suggested that the *MTHFR C677T* locus variation is associated with the occurrence of atrial septal defect (ASD) and patent ductus arteriosus (PDA), and carriers of mutant homozygote *TT* and allele *T* had a high risk of these two types of CHDs [41]. Junker *et al*. studied the relationship of *MTHFR C677T* gene polymorphism with CHDs, and the results showed that *TT* homozygoty was significantly related to congenital heart structure teratogenesis, especially with pulmonary artery stenosis, left heart dysplasia syndrome and coarctation of aorta [35]. Subsequent studies [36–39] failed to show significant results, which could indicate an association between CHD and the *MTHFR* gene polymorphism, which is in consistence to our study but in contrast to the findings of Bennouar *et al*. in a Moroccan population [42].

### 2.3. *C524T* and *A66G* Polymorphisms of *MTRR* Gene

**Figure 2:**
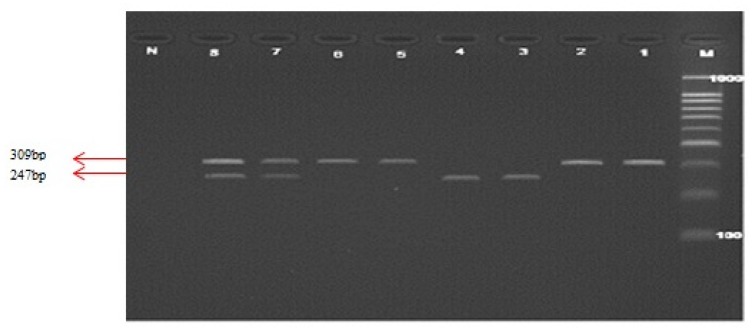
PCR products for methionine synthase reductase (*MTRR) C524T* polymorphism and restricted fragments on 4% agarose gel electrophoresis.

**Figure 3:**
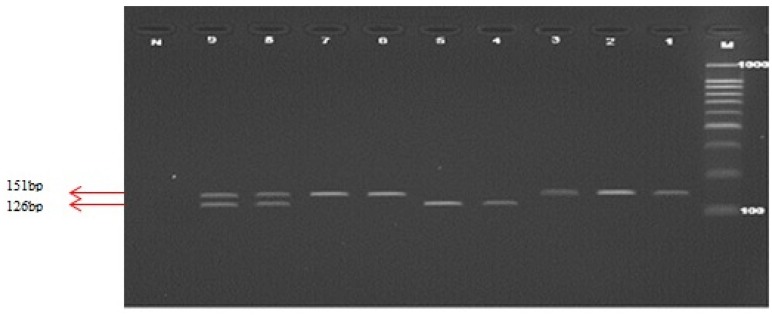
PCR products for *MTRR A66G* polymorphism and restriction fragments on 4% agarose gel electrophoresis.

All genotypes were determined using PCR-RFLP using respective restriction enzymes. In order to genotype *C524T* polymorphism of *MTRR*, the amplified PCR product (as shown in Figure 2) was digested with *Xho1* restriction enzyme. The resolved fragments on 4% agarose gel showed 247 and 62 bp in *C524C* homozygote, 309, 247, and 62 bp in *C524T* heterozygote and 309bp in T524T homozygote. Genotypes of *A66G* polymorphism were determined by digestion of the PCR product with *Nde I* restriction enzyme. This digestion produced fragments of 126, 25 bp for *MTRR* A66A homozygote, 151, 126 and 25 bp in *MTRR A66G* heterozygote, and 151 bp in *MTRR G66G* homozygote. Figure 3 shows the PCR product and RFLP analysis of *MTRR A66G* polymorphism respectively.

In Figure 2, Lane M illustrates the 100 bp DNA ladder. Lanes 1 and 2 show the PCR products (309 bp), Lanes 3 and 4 show the wild type fragments (247 bp), Lanes 5 and 6 show the mutant type fragment (309 bp), Lanes 7 and 8 show the heterozygote fragments (309 and 247 bp) and Lane N represents the negative test control.

In Figure 3, Lane M illustrates the 100 bp DNA ladder. Lanes 1–3 show the PCR products (151 bp), Lane 4,5 shows the wild type fragments (126 bp), Lane 6,7 shows the mutant type fragment (151 bp), Lane 8,9 shows the heterozygote fragments (151 and 126 bp) and Lane N represents the negative test control.

For *MTRR C524T* polymorphism, the percentage of genotypes *C524C*, *C524T* and T524T among cases was 43.1%, 40.7%, and 16.3%, respectively, whereas the frequencies among controls were 52.8%, 43.2% and 4%, respectively (Table 3). There was a significant difference between cases and controls (*p* < 0.05). Derived allele frequencies of C allele and T allele were 63.4% and 36.6% among cases and 74.4% and 25.6% among controls. There was a significant difference between the allele frequency among cases and controls (*p* < 0.05). For the *A66G* polymorphism of *MTRR*, the genotype and frequencies differed significantly between cases and controls (*p* < 0.05). The genotypes of *A66A*, *A66G* and *G66G* were 33.3%, 43.9%, and 22.8%, respectively, in cases compared to 49.6%, 42.4% and 8%, respectively, among control subjects. The allele frequencies as shown in Table 3 also showed a significance difference between cases and controls (*p* < 0.05). *C677T* genotype frequencies were in agreement with those predicted by the Hardy Weinberg distribution, whereas, *MTRR* gene polymorphisms did not meet the Hardy Weinberg assumption in neither cases nor controls.

*MTRR* is a central regulatory enzyme in the metabolic pathway of homocysteine/folate. The malfunction of this enzyme has been considered as a modifiable risk factor for heart and vascular disease [43]. In this case-control study, significant differences were observed for *A66G* and *C524T* polymorphisms of *MTRR* gene between cases and controls (*p* < 0.05), which is in accordance with some studies [44–47]. We found a modest association between the *A66G* and *C524T* alleles of the *MTRR* gene and CHDs in the Iranian subjects. In contrast, a difference in homocysteine concentration between the 66 *MTRR* genotypes in CAD and ischemic cerebrovascular disease was not detected in some studies [14,16]. The conflicting results found in the associated studies were due to many confounding factors, such as study design, sample size bias, gen-environment interactions, population heterogeneity, mismatched phenotypes and population stratification. Collectively, the findings from this study provide adequate evidence that the risks of VSD are influenced by the variants of the three polymorphisms of *MTHFR* and *MTRR* genes in Iranian subjects. It is important to consider the need for future studies of folate-pathway genes other than those found in the current study, and it is also potentially complex to study the gene-gene and gene-environment interactions and VSD in the Iranian population.

### 2.4. DNA Sequencing

For further confirmation, random samples were selected and repeated with the same PCR conditions to confirm the genotyping results. Those samples were purified and DNA sequencing was performed with the Applied Biosystems 3730xl DNA Sequencer provided by Medigene Sdn. Bhd, Selangor, Malaysia.

### 2.5. Study Limitations

In this study, a number of limitations have to be considered. The present study has provided only a genetic association for *MTHFR* and *MTRR* gene polymorphisms among Iranian VSD patients as compared to control subjects. However, we failed to analyze the *MTHFR* and *MTRR* mRNA levels in both VSD patients and control subjects. Apart from the *MTHFR* and *MTRR* gene polymorphisms other polymorphisms such as *TBX5*, *NKX2.5* and *GATA4* need to be analyzed to determine the association of the other candidate genes with VSD and other defects. Replication studies with a larger number of samples and assessing the genotypic and allelic frequencies of mothers are also needed in order to understand the etiological factors.

## 3. Materials and Methods

### 3.1. Ethical Approval

Ethical approval was obtained from the Shaheed Beheshti University of Medical Sciences and also from the Faculty of Medicine and Health Sciences, Universiti Putra Malaysia. A written informed consent was obtained from the parents of the children with CHD. Similarly, consent forms were obtained from all of the control subjects enrolled in this study.

### 3.2. Study Subjects

A total of 150 pediatric patients were approached, and 123 pediatric patients were recruited as the other subjects were excluded due to the inconsistent results and extreme values. All the patients were recruited from the pediatric infectious research center (PIRC), Mofid children hospital, Tehran, Iran. All the subjects were visited during July 2010 to Jun 2011 in the hospital from the different parts of the districts. The diagnosis of non-syndromic CHD and classification of the type of the cardiac defect had been done by a pediatric cardiologist based on the clinical and echocardiography findings with or without the diagnostic cardiac catheterization findings and surgical notes. Whereas for the control subjects, a total of 125 healthy control subjects with no history of congenital heart disease were recruited in for this study.

### 3.3. Genomic DNA Extraction

Buccal cell samples were collected on a sterile cytobrush (Qiagen Inc., Chatsworth, CA, USA) Genomic DNA extraction was carried out using the DNA isolation kit (Qiagen Inc., Chatsworth, CA, USA). The purity of extracted DNA was quantified using Eppendorf UVette® in Biophotometer (Eppendorf, Hamburg, Germany).

### 3.4. Genotyping *MTHFR* and *MTRR* Gene Polymorphisms

To determine the genotypes of *MTHFR* and *MTRR* genes, genomic DNA was amplified first by the respective primers using the polymerase chain reaction (PCR) technique. The PCR amplification for all the respective polymorphisms was performed in a total volume of a 25 μL reaction mixture consisting of 10 pmol of each primer and the Mastermix (i**-**DNA Biotechnology (M) Sdn Bhd, Kuchai lama, Kuala Lumpur, Malaysia) and the template DNA. A negative control containing no genomic DNA and a positive control of known genotype were always included in the set of reactions. All the PCR cycling conditions were carried out on an iCycler machine (BioRad Laboratories, Hercules, CA, USA). The amplified PCR products for all the three gene polymorphisms were separated at 2%–4% agarose gel (Bioline, London, UK). The agarose gel was stained in ethidium bromide and visualized using Alpha Imager (Alpha Innotech, San Leandro, CA, USA). The PCR products of the respective genes were digested with 2–4 units of the respective restriction enzymes (Thermo Fisher Scientific, Inc, provided by Research Instruments Sdn Bhd, Petaling Jaya, Malaysia) with 10× Fast Digest Green Buffer in a final volume of 30 μL reaction mixture. Table 4 shows the primers used for the RFLP method, restriction endonucleases and the digested restricted fragment size products. Identical results were obtained when genotyping was performed for 10% of the samples on two separate occasions.

### 3.5. Automated Sequencing

Purified PCR products were sent to Research Biolabs Malaysia to confirm the nucleotide sequence. The sequencing results were aligned with the respective reference gene sequence from the NCBI-GeneBank sequences using the sequence alignment MEGA4 software [48].

### 3.6. Statistical Analysis

Data analysis was done by SPSS version 18.00 (SPSS Inc, South Wacker Drive, Chicago, IL, USA). Alleles and genotype distribution were tested for deviation from the Hardy-Weinberg by a Chi Square test. The odds ratio (OR) and its 95% confidence intervals (CI) were used to illustrate the association, with *p* < 0.05 considered in all tests to be statistically significant.

## 4. Conclusions

This study failed to show an association between the *MTHFR* gene and VSD subjects. However, the *MTRR* gene polymorphisms (*C524T* and *A66G*) can be considered as risk factors for the development for VSD in Iranian subjects.

## Figures and Tables

**Table 1:** Conflicting results of genetic variants found in different populations.

Gene Variants	Disease	Population/Reference	No. of Subjects	*p*-value
*MTRR* A66G	Vascular disease	Italy [17]	114	NS
*MTRR* A66G	CAD	Morocco [18]	151	S
*MTHFR C677T*	CAD	South Indians [19]	108	S
*MTHFR C677T*	Venous Thrombosis	Iran [20]	200	NS
*MTHFR C677T*	CHD	Japan [21]	233	S
*MTHFR C677T*	CHD	Taiwan [22]	231	S

GATA4	CHD	USA [23]	157	NS
GATA4	CHD	Canada [24]	120	S
GATA4, NKX2.5	CHD	China [25]	62	S
NKX2.5	CHD	China [26]	230	NS
TBX5	VSD	China [1]	192	S

NS: Non-Significant (*p* > 0.05), S: Significant (*p* < 0.05). *MTRR*: methionine synthase reductase; *MTHFR*: methylene tetrahydrofolate reductase; CAD: coronary artery disease; CHD: congenital heart disease.

**Table 2:** Genotypic and allelic distribution of *MTHFR* gene polymorphism.

Gene	Genotypes and Alleles	Case (%)	Control (%)
*C677T* Genotypes	CC	63 (51.2)	71 (56.8)
	CT	60 (48.8)	54 (43.2)
*p* value		0.38 *	
Odds Ratio (95% CI)		0.82 (0.49–1.31)	

Alleles	C	186 (75.6)	196 (78.4)
	T	60 (24.4)	54 (21.6)
*p* value		0.47 *	
Odds Ratio (95% CI)		0.85 (0.56–1.30)	

* *p* value > 0.05.

**Table 3:** Genotypic and allelic distribution of *MTRR* gene polymorphisms.

Gene	Genotypes and Alleles	Case (%)	Control (%)
C524T Genotypes	CC	53 (43.1)	66 (52.8)
	CT	50 (40.6)	54 (43.2)
	TT	20 (16.3)	5 (4.0)
*p* value		0.00 *	

CC *vs.* CT, *p* value		0.60 **	
Odds Ratio (95% CI)		0.87 (0.51–1.47)	
CC *vs.* TT, *p* value		0.00 *	
Odds Ratio (95% CI)		0.20 (0.07–0.57)	
CT *vs.* TT, *p* value		0.00 *	
Odds Ratio (95% CI)		0.23 (0.08–0.66)	

Alleles	C	156 (63.4)	186 (74.4)
	T	90 (36.6)	64 (25.6)
*p* value		0.00 *	
Odds Ratio (95% CI)		0.596 (0.40–0.87)	

**Gene**	**Genotypes and Alleles**	**Case (%)**	**Control (%)**

A66G Genotypes	AA	41 (33.3)	62 (49.6)
	AG	54 (43.9)	53 (42.4)
	GG	28 (22.8)	10 (8.0)
*p* value		0.00 *	

AA *vs.* AG, *p* value		0.12 *	
Odds Ratio (95% CI)		0.65 (0.38–1.12)	
AA *vs.* GG, *p* value		0.00 *	
Odds Ratio (95% CI)		0.24 (0.10–0.54)	
AG *vs.* GG, *p* value		0.00 *	
Odds Ratio (95% CI)		0.36 (0.16–0.82)	

Alleles	A	136 (55.3)	177 (70.8)
	G	110 (44.7)	73 (29.2)
*p* value		0.00 *	
Odds Ratio (95% CI)		0.51 (035–0.73)	

* *p* value < 0.05,

** *p* value > 0.05.

**Table 4:** PCR conditions for the three studied gene polymorphisms using restriction fragment length polymorphism polymerase chain reaction (PCR-RFLP) method.

Gene Polymorphism	Forward Primer (FP) Reverse Primer (RP)	Restriction Endonuclease Enzymes	PCR Products (bp)	Restriction Fragment Size (bp)
*MTHFR C677T*	FP-5′-TGAAGGAGAAGGT GTCTGCGGGA-3′ RP-5′AGGACGGTGCGGT GCGGTGAGAGTG-3′	*Hinf1*	198	Wild type: 198 Mutant: 175, 23 Heterozygote: 198,175, 23
*MTRR* C524T	FP-5′-GTCAAGCAGAGGACA AGAG-3′ RP-5′AGAGACTCCTGCAGAT GTAC-3′	*Xho1*	309	Wild type: 247, 62 Mutant: 309 Heterozygote: 309, 247, 62
*MTRR* A66G	FP-5′-CAGGCAAAGGCCAT CGCAGAAGACAT-3′ RP-5′CACTTCCCAACCAAAA TTCTTCAAAG-3′	*Nde1*	151	Wild type: 126, 25 Mutant: 151 Heterozygote: 151, 126, 25
